# Dopant Control of Solution‐Processed CuI:S for Highly Conductive *p*‐Type Transparent Electrode

**DOI:** 10.1002/advs.202308188

**Published:** 2024-02-01

**Authors:** Minki Son, Ga Hye Kim, Okin Song, ChanHu Park, Sunbum Kwon, Joohoon Kang, Kyunghan Ahn, Myung‐Gil Kim

**Affiliations:** ^1^ School of Advanced Materials Science and Engineering Sungkyunkwan University Suwon 16419 Republic of Korea; ^2^ Department of Chemistry Chung‐Ang University Seoul 06974 Republic of Korea

**Keywords:** copper iodide, metal halide, *p*‐type transparent conductor, solution‐process

## Abstract

Copper iodide (CuI) has garnered considerable attention as a promising alternative to *p*‐type transparent conducting oxides owing to its low cation vacancy formation energy, shallow acceptor level, and readily modifiable conductivity via doping. Although sulfur (S) doping through liquid iodination has exhibited high efficacy in enhancing the conductivity with record high figure of merit (FOM) of 630 00 MΩ^−1^, solution‐processed S‐doped CuI (CuI:S) for low‐cost large area fabrication has yet to be explored. Here, a highly conducting CuI:S thin‐film for *p*‐type transparent conducting electrode (TCE) is reported using low temperature solution‐processing with thiourea derivatives. The optimization of thiourea dopant is determined through a comprehensive acid‐base study, considering the effects of steric hindrance. The modification of active groups of thioureas facilitated a varying carrier concentration range of 9 × 10^18^–2.52 × 10^20^ cm^−3^ and conductivities of 4.4–390.7 S cm^−1^. Consequently, *N*‐ethylthiourea‐doped CuI:S exhibited a FOM value of 7 600 MΩ^−1^, which is the highest value among solution‐processed *p*‐type TCEs to date. Moreover, the formulation of CuI:S solution for highly conductive *p*‐type TCEs can be extended to CuI:S inks, facilitating high‐throughput solution‐processes such as inkjet printing and spray coating.

## Graphical abstract

This work demonstrates the facile solution processing of highly conducting transparent *p*‐type S‐doped CuI with thiourea derivatives as S dopants. Access to the facile solution processing can open the door to high throughput processes like inkjet printing and roll‐to‐roll processing of transparent *p*‐type TCE.

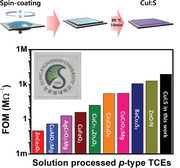

## 1 Introduction

Transparent conductors play a crucial role in large area electronic applications, such as displays, light emitting diodes, and solar cells.^[^ ^1^, ^2^, ^3^, ^4^ ^]^ Although *n*‐type transparent conducting oxides (TCOs), such as indium tin oxide,^[^ ^3^, ^5^ ^]^ fluorine doped tin oxide,^[^ ^6^ ^]^ and aluminum doped zinc oxide,^[^ ^7^ ^]^ have been successfully commercialized with excellent figure of merit (FOM) value exceeding 10^6^ MΩ^−1^, *p*‐type TCO films are plagued by insufficient optoelectronic performance. For typical oxide semiconductors, the localized oxygen 2*p* orbitals at valence band maximum (VBM) and high formation energy of cation vacancies exhibit low hole mobility and suffer from a lack of sufficient hole carriers, respectively. To overcome the limitations, Cu‐based delafossite (CuMO_2_: M = Al, Ga, and In),^[^ ^8^, ^9^ ^]^ ZnM_2_O_4_ (M = Co, Rh, and Ir) spinels,^[^ ^10^, ^11^ ^]^ and layered oxychalcogenides (LaCuOQ: Q = S, Se)^[^ ^12^ ^]^ were recently proposed as *p*‐type TCO candidates with metal *d* – O 2*p* hybridization at VBM. However, the deep defect level, insufficient optical transparency (1.6–3.3 eV),^[^ ^13^ ^]^ and poor hole doping have limited the industrial application of *p*‐type TCOs despite the improved conductivity and FOM values up to 278 S cm^−1^ and 1.1 × 10^4^ MΩ^−1^, respectively.^[^ ^13^ ^]^

Various studies have attempted to obtain an alternative *p*‐type semiconductor system to overcome the challenges of *p*‐type TCOs. Among metal halide semiconductors, copper iodide (CuI) could fulfill the demands of *p*‐type TCEs. Recently, the use of CuI for flexible sensors,^[^ ^14^ ^]^ thermoelectric,^[^ ^15^, ^16^ ^]^ and other flexible electronic devices,^[^ ^17^, ^18^ ^]^ has been demonstrated. Similar to conventional *n*‐type TCOs, CuI with a large bandgap of 3.1 eV is fully transparent for the visible region of 400–800 nm. Owing to the low vacancy formation energy (≈0.085 eV)^[^ ^19^ ^]^ of the copper cation, the defect state of a copper vacancy (*V*_Cu_) is 0.022 eV above VBM of CuI,^[^ ^19^ ^]^ which is significantly shallower than the acceptor energy level of typical *p*‐type oxides (0.7 eV).^[^ ^20^ ^]^ Furthermore, in contrast to oxide semiconductors, Cu 3*d* – I 5*p* hybridization and pseudo *s*‐orbital like characteristic of large I 5*p* orbital provide sufficient band dispersion at the VBM and facile hole conduction with low hole effective mass of 0.3 m_e_.^[^ ^21^ ^]^ With promising intrinsic electrical and optical properties, CuI can open further possibilities of future *p*‐type large area electronics.

Various approaches have been used to deposit intrinsic CuI film, including solid iodination,^[^ ^22^ ^]^ vapor iodination,^[^ ^23^ ^]^ liquid iodination,^[^ ^24^ ^]^ solution‐process,^[^ ^25^ ^]^ and physical deposition methods (pulsed laser deposition, sputtering, and thermal evaporation).^[^ ^16^, ^26^, ^27^, ^28^ ^]^ Among these processes, liquid iodination and solution‐process are suitable for obtaining high quality CuI thin‐film with conductivity of 12–30 S cm^−1^.^[^ ^24^ ^]^ In addition, these processes demonstrate that electrical conductivity of CuI can be improved by doping CuI with ions such as Cs^+^ and S^2−^.^[^ ^24^, ^29^ ^]^ In particular, liquid iodination of Cu with thiol additive has achieved efficient sulfur (S) doping in CuI with record high optoelectronic performances, such as high conductivity of 511 S cm^−1^, hole mobility of 9.8 cm^2^ V^−1^ s^−1^, hole concentration of 3.2 × 10^20^ cm^−3^, and a FOM of 63 000 MΩ^−1^.^[^ ^24^ ^]^ However, the strong chemical binding of S^2−^ to Cu^+^ and poor solubility of S in CuI can hinder the realization of facile S doping in CuI with soluble precursor and cost‐effective fabrication of *p*‐type TCE via a high‐throughput solution‐processes (spin‐coating, spray coating, inkjet printing, and roll‐to‐roll printing).

This study proposed a low‐temperature solution‐processed S‐doped CuI (CuI:S) as a highly conductive *p*‐type TCE, using CuI and thiourea derivatives as S dopant precursors. The effective substitutional doping of S into I and subsequent heavy hole doping of CuI:S was achieved by optimizing the steric effect and basicity of thiourea derivatives. Among the various thiourea derivatives, *N*‐ethylthiourea exhibited the most efficient doping for CuI with the highest hole carrier concentration (*n*_h_) of 2.52 × 10^20^ cm^−3^ and marginal carbon (C) contamination. Along with the investigation of doping concentration and annealing temperature, CuI:S with 1.5% *N*‐ethylthiourea doping and 80 °C annealing exhibited an electrical conductivity of 390.7 S cm^−1^ and a FOM of 7 600 MΩ^−1^, the highest value among solution‐processed *p*‐type TCEs to date.

## 2 Results and Discussion

Soft‐processing is crucial to achieve highly conducting solution‐processed *p*‐type CuI:S thin‐film with high hole mobility and large carrier concentration. CuI is vulnerable to the uncontrolled formation of iodine vacancies, which compensate for the generation of holes and hinder effective carrier transport.^[^ ^25^ ^]^ Acetonitrile with a low boiling point of 86 °C has been utilized to achieve low temperature deposition of CuI thin‐film with solution‐process. The X‐ray photoelectron spectroscopy (XPS) spectrum of N 1*s* in Figure S1 (Supporting Information) confirms that acetonitrile could be clearly removed even at room temperature from the solution‐processed CuI and CuI:S thin‐films similar to previous reports.^[^ ^25^ ^]^ In this study, we employed acetonitrile as a processing solvent of solution‐processed CuI and CuI:S films.

Although the as‐prepared CuI thin‐film exhibit moderate hole concentrations of 10^18^–10^19^ cm^−3^, further improvement of hole concentration is critical to obtain a sufficiently high hole concentration to function as a *p*‐type TCE.^[^ ^24^, ^25^ ^]^ To achieve heavily hole doped CuI, the successful incorporation of S into CuI solution is also critical.^[^ ^24^ ^]^ However, the direct incorporation of S^2−^ in CuI solution could result in immediate precipitation of Cu_2_S with strong interaction between soft acid (Cu^+^) and soft base (S^2−^) even at room temperature.

**Figure 1:**
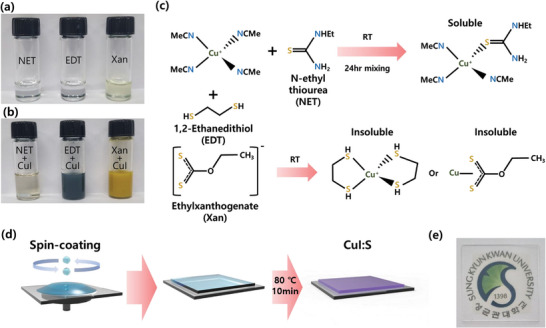
Dopant design and solution‐processing of CuI:S. Photographs of a) as‐prepared dopant solutions and b) CuI and dopant mixed solutions (N‐ethylthiourea, 1,2‐ethanedithiol, and potassium ethylxanthogenate from left to the right). c) Reaction scheme between CuI and various dopants. d) Schematic of spin‐coating process of CuI:S thin‐film. e) Photograph of a *p*‐type transparent conducting CuI:S thin‐film on glass substrate. The university emblem is authorized to be used by Sungkyunkwan University.

As control of the C─S bond strength could allow for a stable metal chalcogenide precursor in the form of chalcogel,^[^ ^30^ ^]^ we initially investigated thiourea, thiol, and xanthogenate, for use as S dopants of CuI in acetonitrile considering a variation of the C─S bond order. As shown in **Figure** **1**a, the representative S dopants of *N*‐ethylthiourea (NET), 1,2‐ethanedithiol (EDT), and potassium ethylxanthogenate (Xan) were clearly soluble in dimethylsulfoxide (DMSO) at 0.05 _M_. After mixing the dopant solutions with 0.144 m CuI in acetonitrile, NET based CuI:S precursor solution in Figure 1b was only sufficiently stable over 24 h. Since acetonitrile is weak binding ligand to Cu^+^ with typical coordination number of four,^[^ ^31^, ^32^ ^]^ the anionic S on EDT, and Xan could easily replace acetonitrile and subsequently form insoluble copper thiolate or copper xanthogenate, as shown in Figure 1c.^[^ ^33^ ^]^ In contrast, the neutral S on NET with stable C═S double bond prevents facile precursor decomposition into Cu_2_S or insoluble adduct formation in the case of the complete removal of acetonitrile from the coordination sphere. As shown in Figure 1d,e, the transparent *p*‐type conducting CuI:S thin‐film was fabricated with spin‐coating of NET containing CuI solution and subsequent mild annealing to decompose the S precursor without significant I vacancy (V_I_) generation.

The ideal S dopant precursor of CuI:S should employ S^2−^ as efficiently as possible without C impurity to achieve optimized hole doping of solution‐processed CuI. The formation of a Cu^+^─S dopant complex and the subsequent formation of CuI:S during the solution process can be interpreted as an acid‐base reaction. Controlling the basicity of the dopant molecules can change the bonding strength between Cu^+^ and dopant molecules. Therefore, modulating the basicity of thiourea species can increase the doping efficiency via the simple adjustment of alkyl chain groups attached to the molecules owing to inductive effects. Although the long alkyl chain or multiple alkyl substitution could enhance the basicity of thiourea derivative and subsequent bonding strength, an increase in C content could result in significant C contamination, as mentioned earlier. Thus, we investigated the structural and chemical properties of CuI:S film with diverse derivatives of thiourea at various concentrations to achieve effective S doping into CuI with minimal impurity incorporation. Exploiting the secured stability of NET doped CuI:S solution, a wide range of alkyl‐thiourea with different chain length, and number were considered for the solution‐process. Thiourea (TU), *N*‐methylthiourea (NMT), *N*‐propylthiourea (NPT), *N*‐isopropylthiourea (NiPT), *N*‐butylthiourea (NBT), *N*‐tertiarybutylthiourea (NtBT), *N*,*N*’‐dimethylthioruea (DMT), and tetramethylthiourea (TMT) were employed as possible candidates of S dopant.

**Figure 2:**
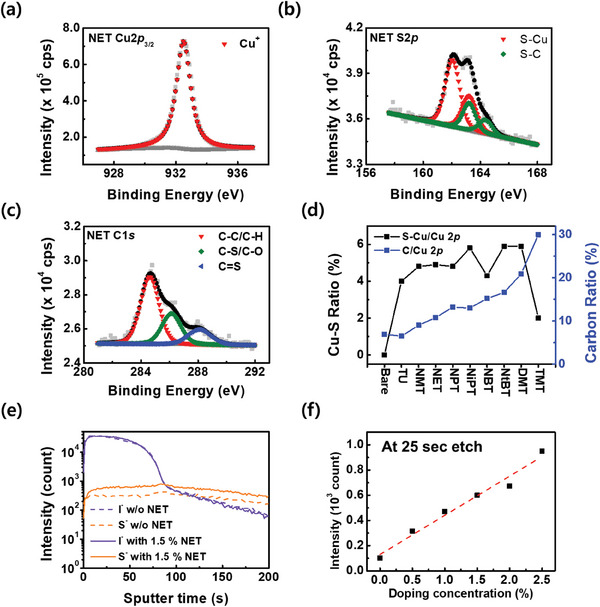
Chemical characterization results of CuI:S films. a) Cu2*p* spectrum, b) S2*p* spectrum, and c) C1*s* spectrum of 10% NET doped CuI:S obtained by X‐ray photoelectron spectroscopy (XPS). d) atomic ratios of S─Cu/Cu and C/Cu of CuI:S (10% doping) obtained by XPS. e) I^−^ signal and S^−^ of 1.5% NET doped CuI:S and bare CuI from time‐of‐flight secondary ion mass spectrometry (ToF‐SIMS). f) Comparison of S^−^ signal obtained by ToF‐SIMS between bare CuI and 0.5%–2.5% NET doped CuI:S at 25 s etching.

The chemical properties of CuI:S thin‐films formed using various dopants were analyzed through XPS. **Figure** **2**a shows the XPS spectrum of Cu2*p* for 10% NET doped CuI:S. As evident, only Cu^+^ signal without mixed oxidation state was observed. S2*p* and C1*s* spectrum were investigated to compare and evaluate the chemical binding differences of CuI:S films with various dopants (Figure 2b,c). From overall XPS analysis of CuI:S films with various dopants at 10% doping, Figure 2d and Table S2 (Supporting Information) present the atomic ratios of C/Cu and S─Cu/Cu. With an increase in the numbers of C atoms in monoalkyl‐substituted thioureas, the C/Cu ratio of CuI:S continuous increased from 6.9% in bare CuI films to 16.6% in NtBT‐doped CuI:S films. Moreover, when compared to NET and NBT, thioureas substituted with multiple alkyl groups and having the same number of C atoms, such as DMT and TMT, exhibited even higher C/Cu ratios of 20.9% and 30.0%, respectively. However, as the basicity of thiourea derivatives increased with the number of C atoms, the atomic ratio of Cu─S/Cu increased, reaching up to 4.8% for NMT and 4.9% for NET. Despite a further increase in the atomic ratio of Cu─S/Cu for NiPT and NtBT up to 5.8%, the significant increase in the C impurity could deteriorate the overall electrical performance of CuI:S films. To identify the S content inside the NET doped CuI:S films, time‐of‐flight secondary ion mass spectrometry (ToF‐SIMS) was conducted. In Figure 2e,f, the comparison of S^−^ and I^−^ signals between bare CuI and 1.5% NET doped CuI:S exhibited an increase in the S^−^ signal with the thiourea dopant. Figure 2f confirms a linear increase of S^−^ signals corresponding to the increase in doping concentration.

**Figure 3:**
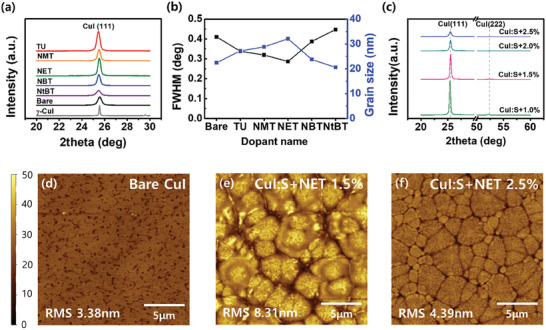
Structural information of CuI:S films. a) θ–2θ X‐ray diffraction (XRD) analysis of bare CuI, TU, NMT, NET, NBT, and NtBT doped CuI:S thin‐films (1.5% doping). b) Full width half maxima (FWHM) values and calculated average grain size of bare CuI, CuI:S doped with TU, NMT, NET, NBT, and NtBT. c) XRD analysis of 1.0%, 1.5%, 2.0%, and 2.5% NET doped CuI:S. Topographic images of d) bare CuI, f) 1.5%, and g) 2.5% NET doped CuI:S obtained with atomic force microscope (AFM).

**Figure** **3**a shows θ–2θ X‐ray diffraction (XRD) results of bare CuI, TU, NMT, NET, NBT, and NtBT doped CuI:S thin‐films (1.5% doping). As shown in Figure S5 (Supporting Information), the solution‐processed CuI:S films only exhibited CuI (111) and CuI (222) signals, indicating that the solution‐processed CuI:S thin‐films were highly textured along the (111) direction. Figure 3b shows the corresponding full width half maxima (FWHM) of the results of Figure 3a and calculated grain size through Scherrer equation. Grain size of the films were 22.4, 27.2, 28.8, 32.1, 23.8, and 20.6 nm for bare CuI, TU, NMT, NET, NBT, and NtBT doped CuI:S with 1.5% doping concentration, respectively. Interestingly, most of the CuI:S films except NtBT doped CuI:S exhibited larger grain size and higher crystallinity than bare CuI without doping, which could be attributed to the seeding effect of Cu^+^‐dopant complexes.^[^ ^34^ ^]^ However, the grain size peak of NET doped CuI:S was observed at 32.1 nm and then it began to decrease. This is because excessively generated C residue cancels out the nucleation effect. In addition, when the doping concentration exceeded 1.5%, the apparent degradation of crystallinity was observed, as shown in Figure 3c. This was attributed to the increase in C residue owing to excess amount of dopant molecules. This is generally common for all dopants, as shown in Figure S5 (Supporting Information). Along with the increase in S content, topographic changes are shown in Figures 3d–f. The bare CuI film in Figure 3d exhibited relatively smooth surfaces with 3.38 nm RMS roughness owing to simultaneous seeding from acetonitrile solution. However, as shown in Figure 3e, apparent seeding behavior with increased RMS roughness of 8.31 nm was observed even with the small S doping of 1.5%. Nevertheless, the collapse of this structure was observed when the doping concentration exceeded 1.5%, as shown in Figure 3f and Figure S7 (Supporting Information). This point was identical to the degradation point of crystallinity shown in Figure 3c.

**Figure 4:**
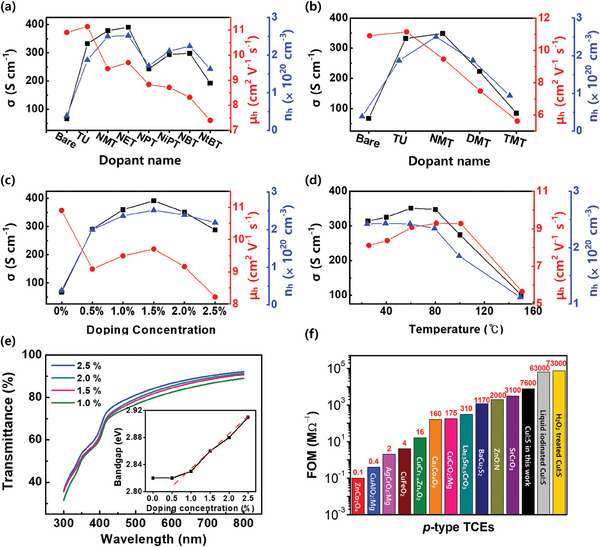
Electrical and optical properties of CuI:S films. a) Electrical properties of CuI:S doped with 1.5% monoalkyl‐substituted thiourea derivatives. b) Electrical property comparison of CuI:S between multi‐alkyl substituted thiourea derivatives (1.5% doping). c) Electrical properties of bare CuI and NET doped CuI:S with different doping concentrations. d) Temperature scan of 1.5% NET doped CuI:S. e) UV–vis spectra and estimated bandgap (inset) of CuI:S films with variation of NET doping concentration. f) Comparison of figure of merit (FOM) with other *p*‐type TCEs (ZnCo_2_O_4_,^[^ ^35^ ^]^ Mg doped CuAlO_2_,^[^ ^36^ ^]^ Mg doped AgCrO_2_,^[^ ^37^ ^]^ CuFeO_2_,^[^ ^38^ ^]^ CuCr_1‐x_Zn_x_O_2_,^[^ ^39^ ^]^ Ca_3_Co_4_O_9_,^[^ ^40^ ^]^ Mg doped CuCrO_2_,^[^ ^41^ ^]^ La_0.5_Sr_0.5_CrO_3_,^[^ ^42^ ^]^ BaCu_2_S_2_,^[^ ^43^ ^]^ N doped ZnO,^[^ ^44^ ^]^ SrCrO_3_,^[^ ^42^ ^]^ and CuI:S in this work and previous work.^[^ ^24^ ^]^).

The electrical and optical properties of CuI:S films with various thiourea dopants were investigated via Hall measurement and UV–vis. For the Hall measurements, **Figure** **4**a and Table S3 (Supporting Information) show the conductivity (σ), hole carrier concentration (*n_h_*), and hole mobility (*µ*_h_) of bare CuI film and mono‐alkyl thiourea doped CuI:S films with 1.5% doping. The bare CuI exhibited *n_h_* of 3.87 × 10^19^ cm^−3^, and the dopants of thiourea (TU) and its derivatives exhibited efficient hole doping up to 2.52 × 10^20^ cm^−3^. The increase in basicity of dopants with linear alkyl group from TU and NMT to NET resulted in a clear improvement of hole doping from 1.87 × 10^20^ cm^−3^ for TU, 2.49 × 10^20^ cm^−3^ for NMT, to 2.52 × 10^20^ cm^−3^ for NET. As reported, hole carrier of pristine CuI originate from copper vacancies (*V*_Cu_) inside the CuI lattice; thus, the defect state of *V*_Cu_ is formed near the VBM.^[^ ^19^ ^]^ S^2−^ defects, which are the source of excessive hole carrier of CuI:S, also formed defect states near VBM and these dense defect states temporally provide a good conduction path.^[^ ^45^ ^]^ As shown in Figure 2b, the effective binding of NET to Cu^+^ with higher basicity facilitated S incorporation and subsequent improvement in the hole doping efficiency. Although *µ*_h_ slightly degraded from 11.14 to 9.70 cm^2^ V^−1^ owing to the increase in C residue, the significant increase in the hole doping improved σ from 332.3 to 390.7 S cm^−1^. However, despite the higher basicity of NPT and NBT, the further increase in the alkyl chain length degraded the hole doping to 1.72 × 10^20^ cm^−3^ for NPT and 2.24 × 10^20^ cm^−3^ for NBT. The longer alkyl chain hindered stable binding of dopant to Cu^+^ and subsequent poor doping despite their higher basicity. Furthermore, residual C impurity from the long alkyl chain significantly degraded the crystallinity that entails decrease of *µ*_h_ from 11.14 to 7.41 cm^2^ V^−1^ s^−1^. Consequently, NET doped CuI:S exhibited the optimized electrical performance of σ = 390.7 S cm^−1^ among all dopants. Compared to TU and mono‐methyl substituted NMT with *n_h_* of 1.87 × 10^20^ and 2.50 × 10^20^, respectively, di‐ and tetra‐methyl substituted DMT and TMT caused sharp degradation of hole doping with *n_h_* of 1.81 × 10^20^ and 9.40 × 10^19^ cm^−3^. S2*p* XPS spectra of DMT and TMT doped CuI in Figure S2 (Supporting Information) shows the SO_3_ and SO_4_ signals. Thus, a majority of DMT and TMT did not form a stable S─Cu bond, and subsequently reacted with moisture upon exposure to the atmosphere.^[^ ^46^, ^47^ ^]^ Although the increase in basicity with multiple alkyl group substitution was expected, the result again confirmed that an excessive increase in the number of alkyl groups on S dopants could result in considerable steric hindrance, thus adversely affecting dopant binding to Cu^+^ ions, and resulting in poor hole doping. Moreover, similar to single alkyl chain length increase, the increase in the number of methyl groups also resulted in C residue and subsequently decreased *µ*_h_ from 11.14 cm^2^ V^−1^ s^−1^ for thiourea, 9.46 cm^2^ V^−1^ s^−1^ for NMT, 7.48 cm^2^ V^−1^ s^−1^ for DMT to 5.6 cm^2^ V^−1^ s^−1^ for TMT. Consequently, 1.5% TMT doped CuI:S exhibited similar conductivity of 84.4 S cm^−1^, compared to that of bare CuI. Even with mono substitution on thiourea, the bulky alkyl group on S dopants, such as isopropyl, and tertiary butyl group, caused significant C residue and steric hindrance to the dopant binding to Cu^+^. The NiPT and NtBT doped CuI:S exhibited dramatic degradation of *µ*_h_ and *n_h_* to 8.71 cm^2^ V^−1^ s^−1^ and 2.11 × 10^20^ cm^−3^ for NiPT and 7.40 cm^2^ V^−1^ s^−1^ and 1.63 × 10^20^ cm^−3^ for NtBT, respectively. The degradation in the electrical performance was harsher with higher doping concentration for bulky or multiple alkyl group substituted S dopants, such as NtBT and TMT. If doping concentration of NtBT or TMT exceeded 1.5%, *µ*_h_ reduced from 8.06 to 2.38 cm^2^ V^−1^ s^−^^1^ for 2.5% NtBT doping. In case of TMT doped CuI:S, the conductivity at 2.5% doping concentration could not be measured.

Based on the Hall measurement and spectroscopic analysis, NET was the optimized *p*‐type dopant of solution‐processed CuI with sufficient basicity and minimal C residues. The optimization of the NET doped CuI:S are shown in Figure 4c with the increase in dopant concentration from 0 to 2.5% and the subsequent change of electrical properties. Bare CuI without any dopants exhibited *n_h_* = 3.87 × 10^19^ cm^−3^, *µ*_h_ = 10.9 cm^2^ V^−1^ s^−1^, and σ = 67.5 S cm^−1^. Further, *n_h_* increased to 2.52 × 10^20^ cm^−3^ until 1.5% NET doping and decreased thereafter. Thus, NET functioned as a dopant until 1.5% doping concentration; however, excess amount of NET becomes molecular scale impurities. In general, although µ_h_ should be decreased along with the substitutional impurity addition, the slight increase of *µ*_h_ from 9.09 to 9.70 cm^2^ V^−1^ s^−1^ until 1.5% doping concentration is attributed to the higher crystallinity with dopant addition, which is well matched with Figure 2e,f. The unusual phenomenon where *n_h_* and µ_h_ both increase simultaneously can be attributed to the fact that, in polycrystalline samples with high concentration of impurities, the mobility of carrier follows either the percolation model or the variable range hopping model.^[^ ^48^, ^49^, ^50^ ^]^ Furthermore, doping of S atoms is more spontaneous to passivate I^−^ vacancies rather than exchanging I^−^ with S^2−^. Nevertheless, doping concentration exceeding 1.5% exhibited *µ*_h_ decrease to 8.22 cm^2^ V^−1^ s^−1^ owing to the increase in residual C. Finally, to optimize the annealing temperature, 1.5% NET doped CuI:S thin‐films were annealed at different temperatures (25–200 °C), as shown in Figure 4d. When processing temperature reached 100 °C over the optimized temperature of 80 °C, *n_h_* decreased from 2.43 × 10^20^ to 1.85 × 10^20^ cm^−3^ and *µ*_h_ increased from 8.06 to 9.28 cm^2^ V^−1^ s^−1^. Thus, *V*_Cu_ of CuI:S decreased owing to low temperature annealing. However, an instant drop for both *µ*_h_ and *n_h_* were observed when annealing temperature exceeded 100 °C owing to the V_I_ formation.^[^ ^25^ ^]^

Figure 4e shows the characterization of optical properties by ultraviolet‐visible spectroscopy (UV–vis). The transparency of films was calculated by averaging transmittance values between λ = 400–800 nm. NET doped CuI:S fabricated by solution‐process exhibited high optical transmittance of 85.03% in the visible range. The bandgap of the CuI:S films was estimated by using standard Tauc equation.^[^ ^24^ ^]^ As interpolated in Figure 4e, it was possible to observe the Burstein‐Moss effect, wherein an increase in doping concentration resulted in an increased optical bandgap.^[^ ^51^ ^]^ Based on the electrical and optical properties of solution‐processed CuI:S film, the FOM was calculated to evaluate, and compare optoelectronic performance. In principle, FOM can be defined as $\frac{\sigma }{\alpha }$ where $\alpha \;=\frac{1}{t}\;\ln\left[\frac{{\left(1-R\right)}^{2}}{T_{vis}}\right]$, with *R, t*, and *T*_vis_ represent the reflectance, thickness, and visible transmittance, respectively. According to Figure 4f, solution‐processed CuI:S in this study exhibited an FOM value of 7 600 MΩ^−1^. Although the FOM is inferior to liquid iodinated *p*‐type CuI:S (73 000 MΩ^−1^),^[^ ^24^ ^]^ RF‐sputtered ITO (FOM = 530 000 MΩ^−1^),^[^ ^52^ ^]^ and solution‐processed ITO (FOM = 320 000 MΩ^−1^),^[^ ^53^ ^]^ it is still bigger than other *p*‐type oxides and a record high value among solution‐processed *p*‐type inorganic TCEs including La_0.5_Sr_0.5_CrO_3_, N‐doped ZnO, BaCu_2_S_2_, and SrCrO_3_.^[^ ^42^, ^43^, ^44^ ^]^ Furthermore, the recent report on solution‐processed CuI:S with elemental sulfur exhibited inefficient hole doping with *n*_h_ of 2.1 × 10^19^ cm^−3^.^[^ ^54^ ^]^ Overall, compared to conventional *p*‐type materials, CuI:S remains a highly prospective materials as shown in recent references.^[^ ^54^, ^55^ ^]^

## 3 Conclusion

Various types of thiourea derivatives were investigated as a S dopant for CuI:S thin‐film fabrication with solution‐process. The simple mixing of CuI solution with dopant solution facilitated the control of the electrical properties of CuI:S films. XPS analysis of S2*p* and C1*s* confirmed that the residual C content and the concentration of doped S varied depending on the functional group attached to thiourea derivatives. ToF‐SIMS spectra of CuI:S confirmed the linear increase in S doping between 0 and 2.5%. Furthermore, XRD confirmed that the highly crystalline CuI:S thin‐film aligned along the (111) direction and crystallinity changed according to the change in the dopant and doping concentration. Topographic images from AFM again verified the crystallinity change with doping concentration. Additionally, UV–vis spectroscopy confirmed the optical transparency of CuI:S films. Carrier concentration measured by Hall measurement demonstrated the effects of basicity and steric hindrance of dopant molecule to hole doping efficiency. Finally, *N*‐ethylthiourea was selected as the most suitable dopant to obtain highly conducting transparent *p*‐type CuI:S films. The optimized processing conditions for *N*‐ethylthiourea‐doped CuI:S were 1.5% doping and an annealing temperature of 80 °C. At this condition, we obtained highly conducting *p*‐type CuI:S thin‐film with thickness of 38.7 nm, σ of 390.7 S cm^−1^, and optical transparency of 85.03%. The FOM value of 7 600 MΩ^−1^ from solution‐processed CuI:S was higher than the FOM values of reported oxide *p*‐type TCEs and the highest value among solution‐processed inorganic *p*‐type TCEs to date. The development of a low‐temperature solution‐process to fabricate CuI:S film represents a significant advancement in the field of transparent *p*‐type electronics. With its promising electrical and optical properties, CuI:S can be potentially employed in a wide range of flexible electronic devices, such as sensors and displays. In addition, the scalability of the solution‐process presents opportunities for low‐cost large‐scale manufacturing, including spin‐coating, spray coating, bar coating, inkjet printing, and roll‐to‐roll process. Further research can explore the full potential of CuI:S in these applications and potentially unlock new opportunities in the field of flexible electronics.

## 4 Experimental Section

### Precursor Solution Preparation

All reagents were purchased from Sigma–Aldrich or Alfa Aesar and were used without further purification. A 0.144 m pristine CuI solution was prepared by dissolving CuI (99.999%, Sigma–Aldrich) in acetonitrile (anhydrous, 99.8%, Sigma–Aldrich). Each dopant solution was prepared by dissolving the dopants in dimethylsulfoxide (anhydrous, 99.9% Sigma–Aldrich) at a concentration of 0.05 m. Thiourea (99.99%, Alfa Aesar), *N*‐methylthiourea (97%, Sigma–Aldrich), *N*‐ethylthiourea (99%, Sigma–Aldrich), *N*‐propylthiourea (98%, Alfa Aesar), *N*‐isopropylthiourea (98%, Alfa Aesar), *N*‐butylthiourea (Sigma–Aldrich), *N*‐tertiarybutylthiourea (Sigma–Aldrich), *N*,*N*’‐dimethylthiourea (99%, Sigma–Aldrich), potassium ethylxanthogenate (96%, Sigma–Aldrich), and 1,2‐ethanedithiol (≥98.0%, Sigma–Aldrich) were used as dopants in this experiment. The solution of CuI and dopants were combined in the desired molar ratios (0.5–10%) and stirred for 30 min prior to use.

### Thin‐Film Deposition of CuI:S

25 mm × 25 mm glass substrates were cleaned by sonication in acetone and isopropyl alcohol for 10 min each, and then treated with an O_2_ plasma for 20 min (CUTE, Femtoscience, and Korea). CuI:S precursor solutions with various dopants were spin‐coated onto the substrates at 3000 rpm for 20 s under an Ar atmosphere. Subsequently, the spin‐coated films were annealed on a hot plate under an Ar atmosphere at various annealing temperature conditions (25, 40, 60, 80, 100, 150, and 200 °C) for 10 min.

### Structural and Morphological Characterization

XRD analysis was performed with AERIS X‐ray diffractometer from Malvern Panalytical Ltd with Cu K_α_ (1.540598 Å) source. XPS analysis was conducted using the K‐Alpha from Thermo Fisher Scientific Inc. with Al K_α_ (1486.6 keV) source. An atomic force microscope (XE7, Park systems, and Korea) was used to obtain topographic image and thickness of CuI:S films. Further, time‐of‐flight secondary ion mass spectrometry was conducted with TOF.SIMS 5 (ION‐TOF, Germany).

### Electrical and Optical Characterization

The Hall measurements of CuI:S films were performed using homemade apparatus under ambient conditions with a Keithley 4200‐SCS semiconductor parameter analyzer. The Hall data were obtained with the Van der Pauw method. Optical property of the film was measured by Cary 5000 UV–vis‐NIR spectrophotometer (Agilent Tech, USA).

## Conflict of Interest

The authors declare no conflict of interest.

## Supporting information

Supporting Information

## Data Availability

The data that support the findings of this study are available in the supplementary material of this article.

## References

[1] R. Mahdiyar , M. R. Fadavieslam , Opt. Quantum Electron. 2020, 52, 262.

[2] K. Fleischer , E. Arca , I. V. Shvets , Sol. Energy Mater. Sol. Cells 2012, 101, 262.

[3] R. Latz , K. M. K. Michael , M. S. M. Scherer , Jpn. J. Appl. Phys. 1991, 30, L149.

[4] T. Rembert , C. Battaglia , A. Anders , A. Javey , Adv. Mater. 2015, 27, 6090. 26455916 10.1002/adma.201502159

[5] Y. Ren , P. Liu , R. Liu , Y. Wang , Y. Wei , L. Jin , G. Zhao , J. Alloys Compd. 2022, 893, 162304.

[6] T. Fukano , T. Motohiro , Sol. Energy Mater. Sol. Cells 2004, 82, 567.

[7] X. Jiang , F. L. Wong , M. K. Fung , S. T. Lee , Appl. Phys. Lett. 2003, 83, 1875.

[8] S. Götzendörfer , C. Polenzky , S. Ulrich , P. Löbmann , Thin Solid Films 2009, 518, 1153.

[9] V. Jayalakshmi , R. Murugan , B. Palanivel , J. Alloys Compd. 2005, 388, 19.

[10] M. N. Amini , H. Dixit , R. Saniz , D. Lamoen , B. Partoens , Phys. Chem. Chem. Phys. 2014, 16, 2588. 24382577 10.1039/c3cp53926a

[11] M. Dekkers , G. Rijnders , D. H. A. Blank , Appl. Phys. Lett. 2007, 90, 021903.

[12] H. Hiramatsu , M. Orita , M. Hirano , K. Ueda , H. Hosono , J. Appl. Phys. 2002, 91, 9177.

[13] M. Ahmadi , M. Asemi , M. Ghanaatshoar , Appl. Phys. Lett. 2018, 113, 242101.

[14] H. J. Lee , M.‐S. Park , S. Lee , B.‐J. Kim , K. Hong , ACS Appl. Electron. Mater. 2022, 4, 1232.

[15] B. M. Morais Faustino , D. Gomes , J. Faria , T. Juntunen , G. Gaspar , C. Bianchi , A. Almeida , A. Marques , I. Tittonen , I. Ferreira , Sci. Rep. 2018, 8, 6867. 29720663 10.1038/s41598-018-25106-3 PMC5932081

[16] C. Yang , D. Souchay , M. Kneiß , M. Bogner , H. M. Wei , M. Lorenz , O. Oeckler , G. Benstetter , Y. Q. Fu , M. Grundmann , Nat. Commun. 2017, 8, 16076. 28681842 10.1038/ncomms16076 PMC5504294

[17] A. Bala , P. Pujar , D. Daw , Y. Cho , M. Naqi , H. Cho , S. Gandla , S. Kim , ACS Appl. Electron. Mater. 2022, 4, 3973.

[18] H. Wu , L. Liang , X. Wang , X. Shi , H. Zhang , Y. Pei , W. Li , B. Sun , C. Shen , H. Cao , Appl. Surf. Sci. 2023, 612, 155795.

[19] K. Ahn , M.‐G. Kim , S. Park , B. Ryu , AIP Adv. 2021, 11, 095018.

[20] J. Tate , H. L. Ju , J. C. Moon , A. Zakutayev , A. P. Richard , J. Russell , D. H. Mcintyre , Phys. Rev. B 2009, 80, 165206.

[21] M. Grundmann , F.‐L. Schein , M. Lorenz , T. Böntgen , J. Lenzner , H. Von Wenckstern , Phys. Status Solidi A 2013, 210, 1671.

[22] N. Yamada , R. Ino , Y. Ninomiya , Chem. Mater. 2016, 28, 4971.

[23] G. Lin , F. Zhao , Y. Zhao , D. Zhang , L. Yang , X. Xue , X. Wang , C. Qu , Q. Li , L. Zhang , Materials 2016, 9, 990. 28774111 10.3390/ma9120990 PMC5456966

[24] K. Ahn , G. H. Kim , S.‐J. Kim , J. Kim , G.‐S. Ryu , P. Lee , B. Ryu , J. Y. Cho , Y.‐H. Kim , J. Kang , H. Kim , Y.‐Y. Noh , M.‐G. Kim , Chem. Mater. 2022, 34, 10517.

[25] A. Liu , H. Zhu , W.‐T. Park , S.‐J. Kang , Y. Xu , M.‐G. Kim , Y.‐Y. Noh , Adv. Mater. 2018, 30, 1802379. 10.1002/adma.201802379 29974529

[26] D. K. Kaushik , M. Selvaraj , S. Ramu , A. Subrahmanyam , Sol. Energy Mater. Sol. Cells 2017, 165, 52.

[27] P. M. Sirimanne , M. Rusop , T. Shirata , T. Soga , T. Jimbo , Chem. Phys. Lett. 2002, 366, 485.

[28] C. Yang , M. Kneiß , M. Lorenz , M. Grundmann , Proc. Natl. Acad. Sci. U.S.A. 2016, 113, 12929. 27807139 10.1073/pnas.1613643113 PMC5135336

[29] K. Matsuzaki , N. Tsunoda , Y. Kumagai , Y. Tang , K. Nomura , F. Oba , H. Hosono , J. Am. Chem. Soc. 2022, 144, 16572. 36049089 10.1021/jacs.2c06283

[30] S. M. Kwon , J. K. Won , J.‐W. Jo , J. Kim , H.‐J. Kim , H.‐I. Kwon , J. Kim , S. Ahn , Y.‐H. Kim , M.‐J. Lee , H.‐I. Lee , T. J. Marks , M.‐G. Kim , S. K. Park , Sci. Adv. 2018, 4, eaap9104. 29662951 10.1126/sciadv.aap9104 PMC5898846

[31] H.‐C. Liang , E. Kim , C. D. Incarvito , A. L. Rheingold , K. D. Karlin , Inorg. Chem. 2002, 41, 2209. 11952376 10.1021/ic010816g

[32] I. Persson , J. E. Penner‐Hahn , K. O. Hodgson , Inorg. Chem. 1993, 32, 2497.

[33] A. T. Casey , A. M. Vecchio , J. Coord. Chem. 1988, 16, 375.

[34] F. Qiu , J. Sun , Z. Zhang , T. Shen , H. Liu , J. Qi , Mater. Today Energy 2021, 21, 100837.

[35] H.‐Y. Chen , P.‐C. Chen , Appl. Surf. Sci. 2020, 505, 144460.

[36] H. F. Jiang , X. B. Zhu , H. C. Lei , G. Li , Z. R. Yang , W. H. Song , J. M. Dai , Y. P. Sun , Y. K. Fu , J. Alloys Compd. 2011, 509, 1768.

[37] R. Wei , X. Tang , L. Hu , J. Yang , X. Zhu , W. Song , J. Dai , X. Zhu , Y. Sun , J. Mater. Chem. C 2017, 5, 1885.

[38] H.‐Y. Chen , J.‐H. Wu , Appl. Surf. Sci. 2012, 258, 4844.

[39] H.‐Y. Chen , C.‐C. Yang , Surf. Coat. Technol. 2013, 231, 277.

[40] M. Aksit , S. K. Kolli , I. M. Slauch , R. D. Robinson , Appl. Phys. Lett. 2014, 104, 161901.

[41] T.‐W. Chiu , S.‐W. Tsai , Y.‐P. Wang , K.‐H. Hsu , Ceram. Int. 2012, 38, S673.

[42] K. H. L. Zhang , Y. Du , A. Papadogianni , O. Bierwagen , S. Sallis , L. F. J. Piper , M. E. Bowden , V. Shutthanandan , P. V. Sushko , S. A. Chambers , Adv. Mater. 2015, 27, 5191. 26248327 10.1002/adma.201501959

[43] Y. Wang , M. Liu , F. Huang , L. Chen , H. Li , X. Lin , W. Wang , Y. Xia , Chem. Mater. 2007, 19, 3102.

[44] H. Nian , S. H. Hahn , K.‐K. Koo , E. W. Shin , E. J. Kim , Mater. Lett. 2009, 63, 2246.

[45] M. Grauzinyte , S. Botti , M. A. L. Marques , S. Goedecker , J. A. Flores‐Livas , Phys. Chem. Chem. Phys. 2019, 21, 18839. 31353386 10.1039/c9cp02711d

[46] S. Sahu , P. Rani Sahoo , S. Patel , B. K. Mishra , J. Sulfur Chem. 2011, 32, 171.

[47] S. Wei , C. Ma , X. Liu , N. Liu , M. Yuan , K. Xiao , W. Yan , H. Xin , Chem. Commun. 2023, 59, 9848. 10.1039/d3cc02417b 37489840

[48] G. Paasch , T. Lindner , S. Scheinert , Synth. Met. 2002, 132, 97.

[49] D. Benlakehal , A. Belfedal , Y. Bouizem , J. D. Sib , L. Chahed , K. Zellama , Superlattices Microstruct. 2016, 100, 228.

[50] K. Nomura , H. Ohta , A. Takagi , T. Kamiya , M. Hirano , H. Hosono , Nature 2004, 432, 488. 15565150 10.1038/nature03090

[51] I. Hamberg , C. Granqvist , K.‐F. Berggren , B. Sernelius , L. Engström , Vacuum 1985, 35, 207.

[52] S. Yang , J. Zhong , B. Sun , X. Zeng , W. Luo , X. Zhao , Y. Shu , J. Chen , J. He , J. Mater. Sci.: Mater. Electron. 2019, 30, 13005.

[53] Z. Chen , W. Li , R. Li , Y. Zhang , G. Xu , H. Cheng , Langmuir 2013, 29, 13836. 24117323 10.1021/la4033282

[54] F. Geng , L. Wang , T. Stralka , D. Splith , S. Ruan , J. Yang , L. Yang , G. Gao , L. Xu , M. Lorenz , M. Grundmann , J. Zhu , C. Yang , Adv. Eng. Mater. 2023, 25, 2201666.

[55] A. S. Mirza , M. Pols , W. Soltanpoor , S. Tao , G. Brocks , M. Morales‐Masis , Matter 2023, 6, 4306.

