# Supplementary material for: Dopant Control of Solution‐Processed CuI:S for Highly Conductive p‐Type Transparent Electrode
Source: Adv Sci (Weinh). 2024 Feb 1;11(14):2308188. doi: 10.1002/advs.202308188 (PMC11005697; doi:10.1002/advs.202308188)
Supplement: Supplementary file 1 — Supporting Information [file ADVS-11-2308188-s001.pdf]

ADVANCED SCIENCE
Open Access

Supporting Information

for *Adv. Sci.*, DOI 10.1002/advs.202308188

Dopant Control of Solution-Processed CuI:S for Highly Conductive *p*-Type Transparent Electrode

Minki Son, Ga Hye Kim, Okin Song, ChanHu Park, Sunbum Kwon, Joohoon Kang, Kyunghan Ahn and Myung-Gil Kim**

Supporting Information

Dopant Control of Solution Processed CuI:S for Highly Conducting *p*-Type Transparent Electrodes

Minki Son, Ga Hye Kim, Okin Song, ChanHu Park, Sunbum Kwon, Joohoon Kang, Kyunghan Ahn[*] and Myung-Gil Kim[*]

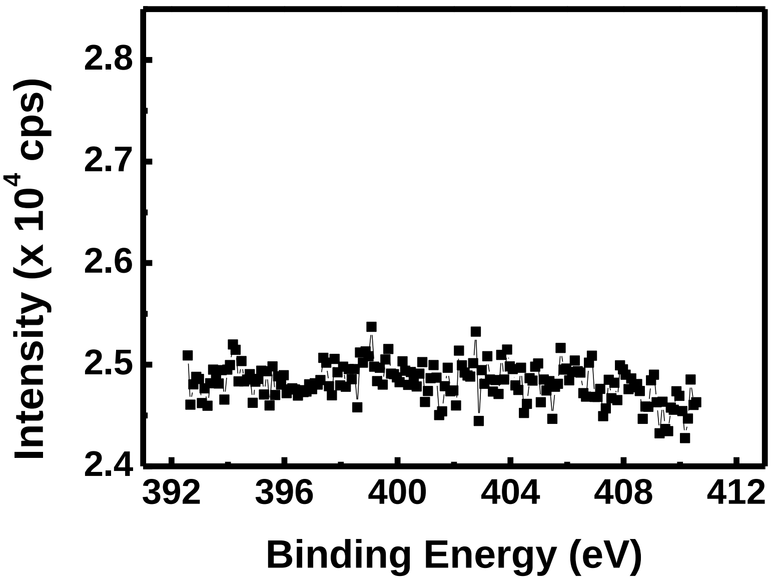

Figure S1. N 1*s* X-ray photoelectron spectrum of bare CuI without doping.

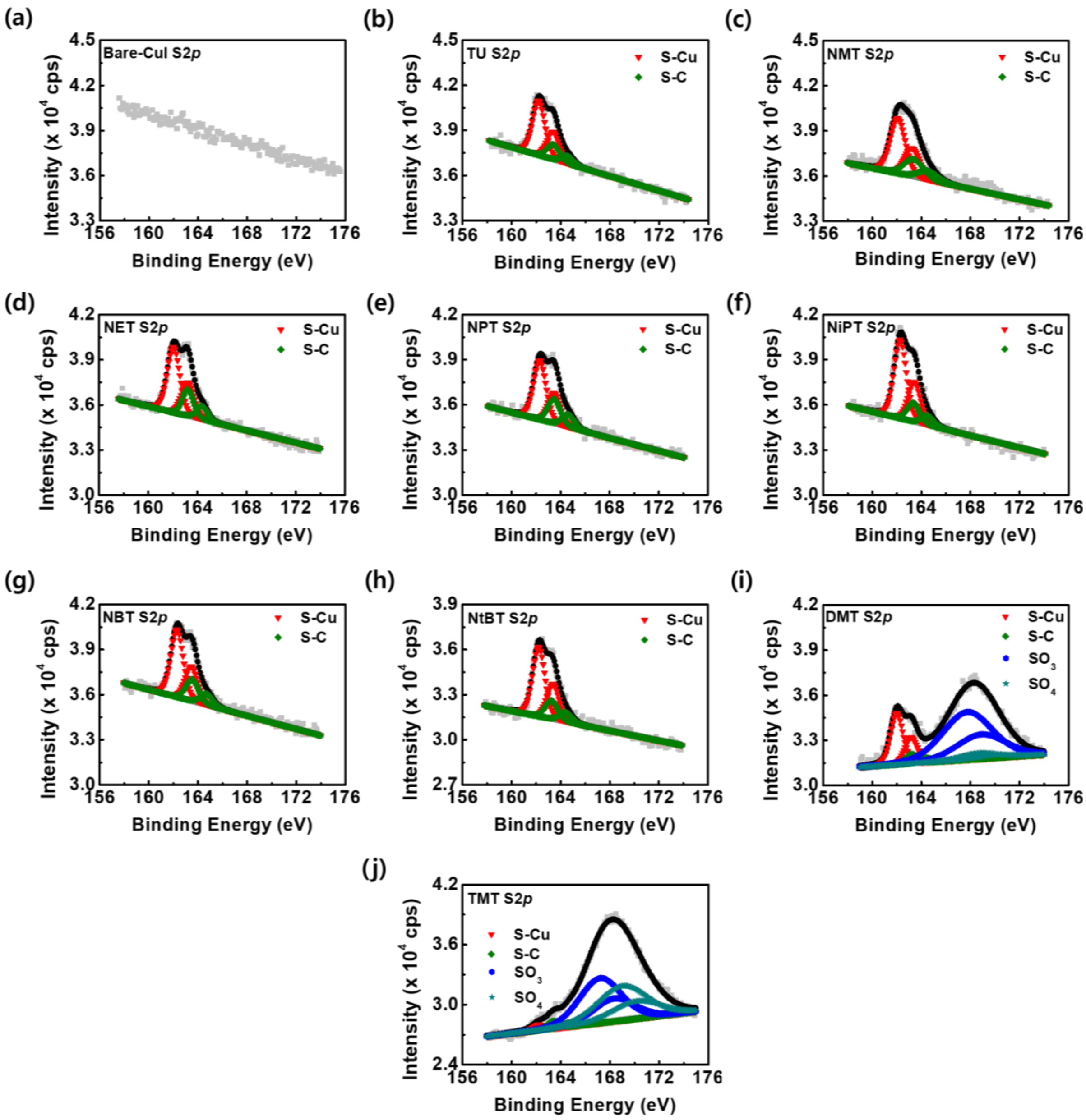

Figure S2. S2*p* XPS spectra of bare CuI and 10 % doped CuI:S with thiourea derivatives.

The SO_3 and SO_4 signals of DMT S2*p* and TMT S2*p* result from the unreacted residual thiourea derivatives reacting with moisture when exposed to the ambient air.

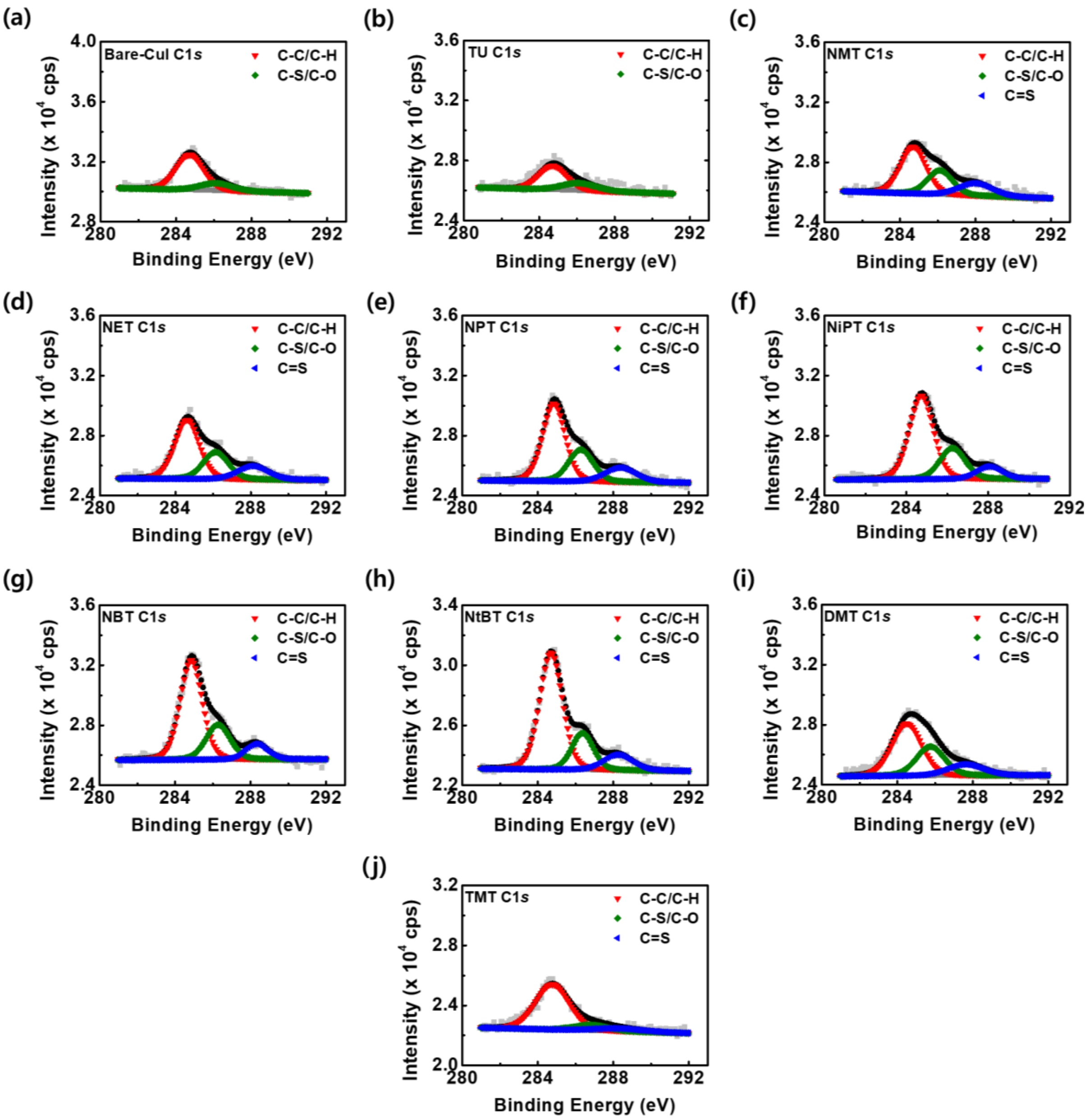

Figure S3. C1*s* XPS spectra of bare CuI and 10 % doped CuI:S with thiourea derivatives.

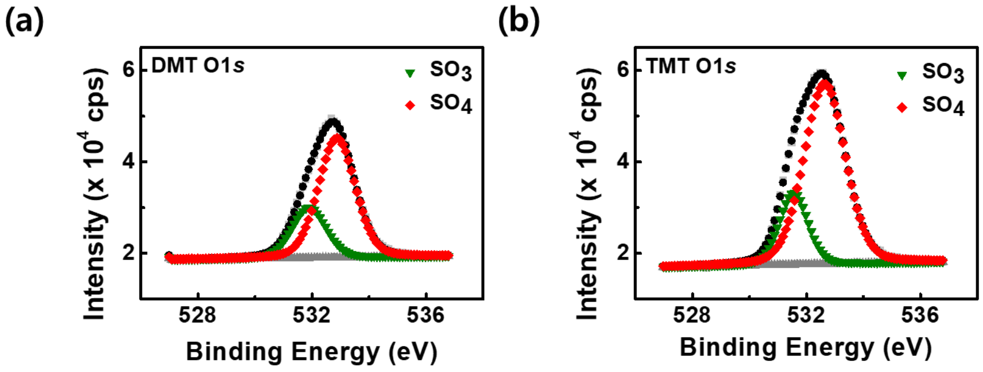

Figure S4. O1*s* XPS spectra of 10 % (a) DMT doped CuI:S and (b) TMT doped CuI:S.

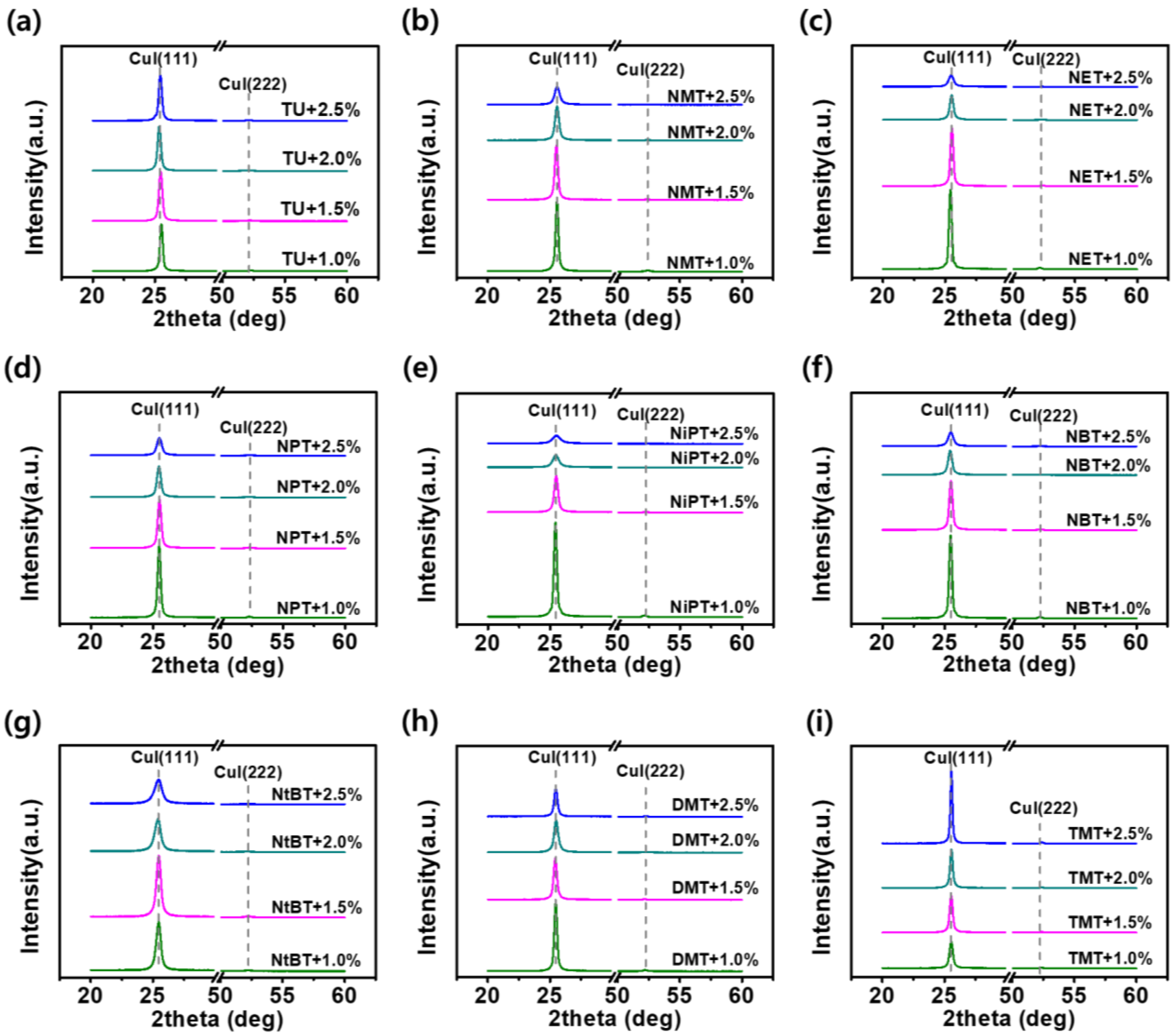

Figure S5. θ-2θ XRD data of various thiourea derivatives doped CuI:S thin-films with different doping concentrations.

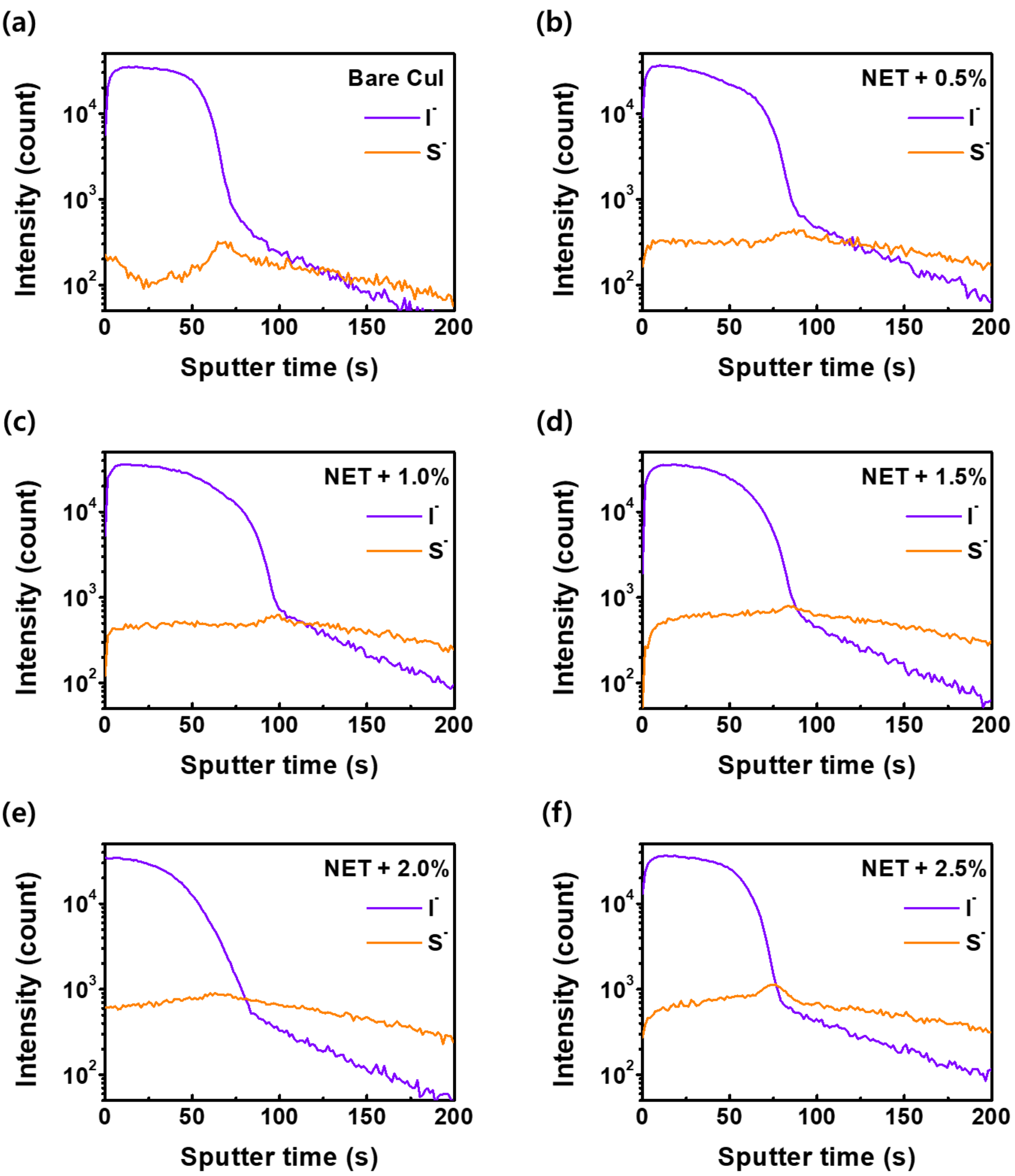

Figure S6. Time of flight secondary ion mass spectroscopy (ToF-SIMS) analysis of a) bare-CuI, b) 0.5 %, c) 1.0 %, d) 1.5 %, e) 2.0 %, and f) 2.5 % NET doped CuI:S. Purple line indicates the I^- signal and orange line indicates the S^- signal.

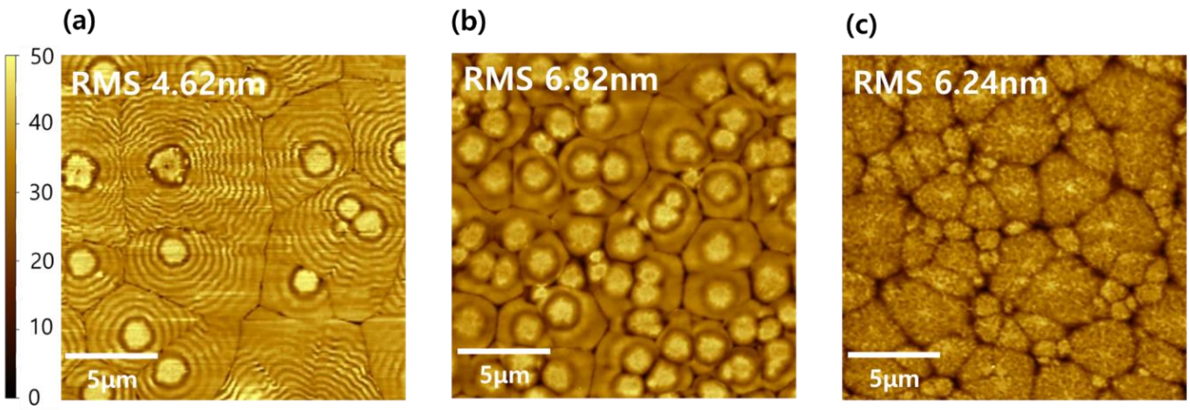

Figure S7. Topographic images of a) 0.5%, b) 1.0 %, and c) 2.0 % NET doped CuI:S obtained with atomic force microscope.

Table S1. Area of S2$p_{3/2}$ XPS deconvoluted from Figure S2. Each number has been rounded to the nearest tenth.

Dopant name	Area of S-Cu	Area of S-C	Area of SO_3	Area of SO_4
none[a]	-	-	-	-
Thiourea	1400	5100	-	-
N-methylthiourea	1900	6000	-	-
N-ethylthiourea	1700	6200	-	-
N-propylthiourea	1700	5600	-	-
N-isopropylthiourea	1500	7200	-	-
N-butylthiourea	1700	6200	-	-
N-(tert-butyl)-thiourea	1300	6800	-	-
N,N'-dimethylthiourea	5000	400	17600	1500
Tetramethylthiourea	800	900	20600	18800

[a]Copper iodide (CuI) without doping

Table S2. Atomic ratios of C/Cu and S-Cu/Cu from XPS analysis of CuI:S with various dopants at 10 % doping

Dopant name	C content (%)	Cu content (%)	Area of S-Cu	Total area of S	C/Cu (%)	S-Cu/Cu (%)
none[a]	3.34	48.43	-	-	6.9	-

Thiourea	3.3	50.66	1400	6500	6.5	4
N-methylthiourea	4.37	48.66	1900	7900	9	4.8
N-ethylthiourea	5.3	49.14	1700	7900	10.8	4.9
N-propylthiourea	6.38	48.34	1700	7300	13.2	4.8
N-isopropylthiourea	6.31	48.64	1500	8700	13	5.8
N-butylthiourea	7.31	48.21	1700	7900	15.2	4.3
N-(tert-butyl)-thiourea	7.87	47.40	1300	8100	16.6	5.9
N,N’-dimethylthiourea	7.65	36.60	5000	24500	20.9	5.9
Tetramethylthiourea	6.85	22.85	800	41100	30	2

[a]Copper iodide (CuI) without doping

In case of the C/Cu ratio, the ratio was calculated by dividing atomic ratio of C obtained from C1*s* spectrum by the atomic ratio of Cu obtained from Cu2*p* spectrum. Further, the intensity of the S-Cu S2$p_{3/2}$ and S-C S2$p_{3/2}$ signals was deconvoluted to separate the S-Cu bonding from total atomic ratio of S as shown in Figure S2 and Table S1. Consequently, S-Cu/Cu atomic ratio was calculated by following equation.

$$(\text{at \% ratio of S-Cu/Cu}) = \frac{\frac{(\text{Intensity of S-Cu S}2p_{3/2}\text{ signal})}{(\text{Total intensity of S}2p_{3/2}\text{ signal})} \times (\text{at \% of S})}{(\text{at \% of Cu})} \times 100$$

Table S3. Summary of Hall measurement data with variations of dopants and doping concentrations.

Dopant name	Doping conc.[%]	Thickness[a] [nm]	Conductivity [S cm^{-1}]	Hall mobility [cm^2 V^{-1} s^{-1}]	Hole conc. [10^{20} cm^{-3}]
Thiourea	1.0	35.11	293.5	9.90	1.85
	1.5	36.92	332.3	11.14	1.87
	2.0	34.26	318.1	10.93	1.82
	2.5	40.02	261.1	10.69	1.53
N-methylthiourea	1.0	41.95	269.5	8.95	1.88
	1.5	40.65	378.6	9.46	2.50
	2.0	43.38	280.7	9.09	1.93

	2.5	38.17	242.4	7.34	2.06
N-ethylthiourea	1.0	41.00	347.4	9.86	2.20
	1.5	38.71	390.7	9.70	2.52
	2.0	39.67	350.5	9.16	2.39
	2.5	40.57	287.6	8.22	2.19
N-propylthiourea	1.0	39.81	247.3	8.59	1.80
	1.5	44.38	242.7	8.83	1.72
	2.0	41.98	221.5	8.09	1.71
	2.5	38.97	176.4	6.75	1.63
N-isopropylthiourea	1.0	43.97	249.1	9.04	1.72
	1.5	41.02	294.1	8.71	2.11
	2.0	38.35	240.9	7.14	2.11
	2.5	42.99	175.6	6.08	1.81
N-butylthiourea	1.0	40.08	291.5	8.87	2.01
	1.5	39.73	298.5	8.32	2.24
	2.0	39.29	214.7	6.87	1.95
	2.5	36.31	175.8	5.35	2.06
N-(tert-butyl)thiourea	1.0	44.23	214.5	8.06	1.66
	1.5	46.25	193.0	7.40	1.63
	2.0	39.87	150.0	4.38	2.14
	2.5	38.78	82.2	2.38	2.16
N,N'-dimethylthiourea	1.0	41.47	187.5	6.80	1.72
	1.5	44.61	223.2	7.48	1.81
	2.0	42.77	305.0	8.35	2.28
	2.5	39.46	249.5	6.30	2.48
Tetramethylthiourea	1.0	35.14	118.8	6.13	1.21
	1.5	40.44	84.4	5.63	0.94
	2.0	48.34	4.4	3.11	0.09
	2.5	66.15	Impossible to measure[b]		

[a]Thickness is measured by AFM

[b]$R_{H,13}$ and $R_{H,24}$ is different severely for all possible current inputs (0.01 A ~ 1×10^{-7} A), which prohibit the reliable Hall measurement.
